# Increased recognition of Q fever aortitis as a chronic manifestation of Q fever in tropical North Queensland, Australia

**DOI:** 10.1007/s10096-023-04687-6

**Published:** 2023-10-26

**Authors:** Cody Price, Simon Smith, James Stewart, Tom Palesy, Matthew Corbitt, Charith Galappaththy, Josh Hanson

**Affiliations:** 1 Department of Medicine, Cairns Hospital, Cairns, Queensland Australia; 2 Department of Surgery, Cairns Hospital, Cairns, Queensland Australia; 3 School of Medicine and Dentistry, Griffith University, Gold Coast, Queensland Australia; 4 Kirby Institute, University of New South Wales, Sydney, Level 6, Wallace Wurth Building High Street, Kensington, New South Wales 2052 Australia

**Keywords:** Q fever, *Coxiella burnetii*, Aortitis, Vascular prosthesis, Prosthesis-related infections, Zoonosis

## Abstract

Aortitis is a life-threatening, manifestation of chronic Q fever. We report a series of 5 patients with Q fever aortitis who have presented to our hospital in tropical Australia since 2019. All diagnoses were confirmed with polymerase chain reaction (PCR) testing of aortic tissue. Only one had a previous diagnosis of acute Q fever, and none had classical high-risk exposures that might increase clinical suspicion for the infection. All patients underwent surgery: one died and 3 had significant complications. Q fever aortitis may be underdiagnosed; clinicians should consider testing for *Coxiella burnetii* in people with aortic pathology in endemic areas.

## Introduction

Q fever — caused by the obligate intracellular bacterium *Coxiella burnetii* — is a zoonotic infection with a worldwide distribution. Over half of acute Q fever infections are subclinical while symptomatic acute infections usually present as an influenza-like illness [1]. This non-specific presentation — combined with many clinicians’ limited awareness of the disease — means that acute Q fever is likely to be underrecognised, particularly if it occurs in people without classical recreational or occupational risk factors [2]. Chronic Q fever — most often manifesting as endocarditis or vascular infection — is believed to complicate approximately 5% of symptomatic acute Q fever cases, but the proportion of individuals with subclinical infection who develop chronic disease is unknown [1, 2].

Q fever is endemic in Australia and is a notifiable disease. Almost half of Australia’s cases occur in the state of Queensland in the country’s northeast [3, 4]. Annual rates fluctuate, but are highest in Mareeba, a farming region in tropical Far North Queensland (FNQ), where the annual incidence approaches 1000/100,000 [3]. Q fever aortitis is a rare, but life-threatening, manifestation of chronic Q fever which is gaining increasing recognition. Indeed, vascular infection is more common than endocarditis in chronic Q fever patients in the Netherlands [5]. We present a series of 5 cases of Q fever aortitis diagnosed in tropical Queensland. Only one had a previous diagnosis of acute Q fever, and none had classical high-risk exposures that might increase clinical suspicion for the infection.

## Materials and methods

This study was performed in Cairns Hospital, the sole tertiary referral hospital in FNQ. Cases of Q fever aortitis in FNQ residents were identified by interrogating the Queensland electronic pathology reporting system (AUSLAB). We reviewed all positive *C. burnetii* polymerase chain reaction (PCR) results between October 2003 and March 2023, a period that coincided with introduction of the test in the public health system. Patients were defined as having Q fever aortitis if *C. burnetii* was detected by PCR in aortic tissue. The patients’ medical charts were reviewed, and their presentation and clinical course recorded. The Far North Queensland Human Research Ethics Committee provided ethical approval for the study (EX/2023/QCH/95302–1707QA). As the retrospective data were de-identified, the Committee waived the requirement for informed consent.

There were 61 positive PCR results from 56 FNQ residents. There were 5 PCR tests on aortic tissue, all 5 of these tests occurred after 2019 and all were positive. The cases all occurred in older males (median (range) 68 (55–76) years) who all lived locally but only one had a history of serologically confirmed acute Q fever. Two patients had presented with fever, thrombocytopaenia and transaminitis — consistent with acute Q fever — 3 and 6 years previously respectively, but neither were tested for Q fever. All cases had a plausible exposure, although none would have been considered high-risk (Table 1) [2]. None of the cases had received Q fever vaccination.

**Table 1:** Overview of the clinical characteristics of five cases of Q fever aortitis

Case	Age/Sex	Occupational or recreational exposure	History of confirmed acute Q fever	Time from confirmed or plausible acute Q fever infection to clinical presentation	Predisposing vascular lesion	Clinical scenario at time of Q fever aortitis diagnosis	Vascular lesion	Aortic FDG avidity ^a^	Outcome
1	70 M	Rural residence	No	Unknown	Yes	Sepsis and known previous AAA graft infection	Polymicrobial infrarenal endovascular aortic graft infection	Not performed	Alive at 3 years
2	68 M	Rural residence, kangaroos on property	No	Unknown	Yes	Abdominal pain, known previous AAA rupture	Ruptured infrarenal AAA post endovascular aortic repair	Not performed	Intra-operative death
3	73 M	Rural residence, hand fed bandicoots	Yes	1 year	Yes	Incidental following acute Q fever hepatitis	Enlarging infrarenal AAA	Yes	Alive at 1 year
4	55 M	Clearing scrub land for construction	No	6 years	Yes	Incidental following out of hospital cardiac arrest	84 mm infrarenal AAA with radiological signs of inflammation	Yes	Alive at 7 months
5	76 M	Rural residence, bull visiting property	No	3 years	Yes	Inflamed aorta during elective open AAA repair	31 mm saccular pararenal AAA	Not performed	Alive at 6 months

^a^Fluorodeoxyglucose avid (FDG) on positron emission tomography

*M*, male; *AAA*, abdominal aortic aneurysm

**Fig. 1:**
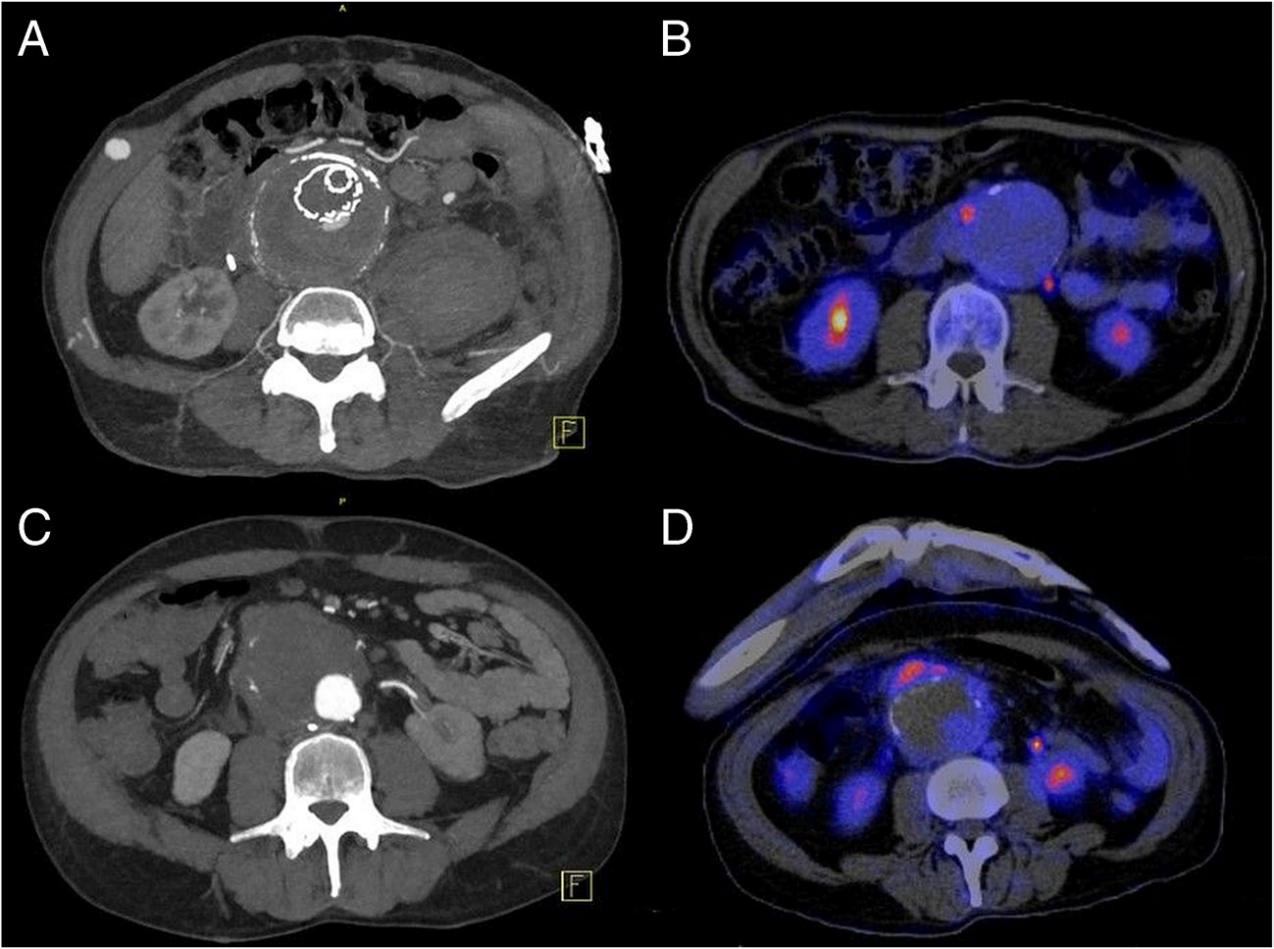
Imaging findings in Q fever aortitis. Panel **A**: Computed tomography angiography (CTA) demonstrating a ruptured aortic aneurysm with vascular endograft in case 2. Panel **B**: Fluorodeoxyglucose avid (FDG) avidity on positron emission tomography (PET) of the aneurysm wall in case 3. Panel **C**: CTA demonstrating 84 mm infrarenal aortic aneurysm with radiological signs of anterior aortic wall inflammation in case 4, with demonstrated FDG avidity in the aneurysm wall on the corresponding PET (panel **D**)

All 5 had a predisposing vascular lesion. In 4, complications of aortic aneurysmal disease preceded their Q fever diagnosis (Table 1). Case 1 presented in 2019 with sepsis and a history of a culture negative aortic graft infection 3 years previously. Q fever had been considered at that time and serology revealed a phase 1 IgG (immunofluorescence) level of 1:320 but he received only 3 months of antibiotics. Explant of the graft in 2019 demonstrated polymicrobial infection including *C. burnetii* (confirmed on PCR), as well as growth of *Escherichia coli*, *Streptococcus milleri*, *Prevotella oris* and *Cutibacterium acnes.* Case 2 presented with a ruptured endovascular aortic repair and raised inflammatory markers that prompted Q fever testing. In case 3, aortic aneurysmal disease was incidentally diagnosed at the time of acute Q fever and subsequent imaging demonstrated an enlarging aneurysm with inflammatory features (Fig. 1). Case 4 had a cardiac arrest from coronary artery disease and was concurrently diagnosed with an infrarenal abdominal aortic aneurysm (AAA) with radiological signs of inflammation (Fig. 1). Case 5 presented electively for open aortoiliac reconstruction for a saccular aortic aneurysm and symptomatic atherosclerotic iliac disease. Q fever testing was prompted by an inflammatory macroscopic appearance of the aneurysm at surgery. Four cases underwent transthoracic echocardiography; none had vegetations or valvular lesions that might predispose to Q fever endocarditis.

All cases underwent aortic surgery. Both infected endovascular graft cases had graft explantation. Of the remainder, two had open aortic repair and one underwent endovascular repair, as open repair was considered too high-risk. One patient died during surgery, one suffered a hypoxic brain injury, one required drainage of a paraaortic collection and one had a post-operative ileus requiring short-term total parenteral nutrition. Aortic tissue was collected at surgery in 4 cases and by imaging-guided aortic biopsy following endovascular repair in one case (Table 2).

**Table 2:** *C. burnetii*-specific diagnostic tests, at time of vascular assessment

Case	IgM (EIA)	IgG phase I titer (IF)	IgG phase II titer (IF)	PCR blood	PCR tissue
1	Negative	1:320	1: ≥ 1280	Negative	Positive
2	Negative	1: ≥ 1280	1: ≥ 1280	Positive	Positive
3	Negative	1: ≥ 1280	1: ≥ 1280	Negative	Positive
4	Negative	1: ≥ 1280	1:320	Negative	Positive
5	Negative	1: ≥ 1280	1: ≥ 1280	Negative	Positive

*EIA*, enzyme immunoassay; *IF*, immunofluorescence; *PCR*, polymerase chain reaction

Antibiotics were prescribed in all cases who survived surgery. One completed 18 months of doxycycline and hydroxychloroquine therapy, one was unable to tolerate doxycycline, hydroxychloroquine or ciprofloxacin and due to intractable nausea and limited alternative options, was prescribed trimethoprim/sulfamethoxazole for 24 months. Two are currently receiving doxycycline and ciprofloxacin for a planned 24 months.

## Discussion

Aortitis is an uncommon manifestation of chronic Q fever; however, this series of 5 cases in 4 years demonstrates that it may occur in people without classical high-risk exposures. In 4 of the cases, there was no documented history of acute Q fever prior to presentation with vascular complications. Furthermore, all but one had negative results on PCR testing of blood, highlighting that a negative blood PCR is not necessarily sufficient to exclude the diagnosis.

Two cases had historical hospital presentations with a febrile illness consistent with Q fever, although neither had appropriate testing. The subsequent life-threatening complications of undiagnosed Q fever highlights the need for an increased awareness of disease and access to sensitive diagnostic testing (including, if necessary, PCR on tissue and fluorodeoxyglucose positron emission tomography-computed tomography scanning) which may expedite its recognition and treatment.

Q fever is preventable. However, vaccination — which is offered only in Australia — is complicated by the requirement for prior serological and skin testing and is therefore reserved for those at highest risk for the disease. None of the patients in our case series had been vaccinated nor did they meet current recommended criteria for vaccination [2]. However, the development of Q fever vaccines which are easier to administer and with fewer adverse effects may allow expansion of vaccination beyond traditional high-risk groups [6].

Following acute Q fever, chronic vascular infection can develop in patients with pre-existing aneurysms or vascular grafts and carries a case-fatality rate of 18–26% [7]. Our series suggests that, in the appropriate clinical context, patients with risk factors for atherosclerosis should be screened for aortic aneurysms at the time of acute Q fever diagnosis as they — and patients with vascular prostheses — may benefit from enhanced follow-up [8]. In endemic regions, screening of patients with aortic aneurysmal disease with Q fever serology may also have utility.

This case series only included PCR-positive cases and may underestimate the burden of disease as aortic tissue was only tested after 2019 [9]. Its retrospective nature means that specific risk factors may not have been documented, although all cases had infectious diseases specialist review.

## Conclusion

All 5 patients with Q fever aortitis in this series had underlying aortic pathology, but all lacked a classical high-risk exposure history. All patients underwent surgery: one died and 3 had significant complications. The series highlights the need for an increased awareness of Q fever, enhanced access to sensitive diagnostic testing, and consideration of Q fever testing of people with aortic pathology who live in areas of high Q fever incidence.

## Data Availability

All relevant data for this case series are presented in the tables of the manuscript.
